# Endometriosis in Menopausal Women—A New Age Is Coming? Literature Review

**DOI:** 10.3390/life14040485

**Published:** 2024-04-09

**Authors:** Mihai-Daniel Dinu, Bashar Haj Hamoud, Mihaela Amza, Gabriel-Petre Gorecki, Romina-Marina Sima, Nicolae Gică, Liana Pleș

**Affiliations:** 1 Department PhD, IOSUD, “Carol Davila” University of Medicine and Pharmacy, 020021 Bucharest, Romania; mihai-daniel.dinu@drd.umfcd.ro (M.-D.D.); mihaela.amza@umfcd.ro (M.A.); 2 Department for Gynecology, Obstetrics and Reproductive Medicine, Saarland University Hospital, Kirrberger Straße 100, Building 9, 66421 Homburg, Germany; bashar.hajhamoud@uks.eu; 3 Department of Obstetrics and Gynecology, “Carol Davila” University of Medicine and Pharmacy, 020021 Bucharest, Romania; gica.nicolae@umfcd.ro (N.G.); liana.ples@umfcd.ro (L.P.); 4 “Bucur” Maternity, Saint John Hospital, 012361 Bucharest, Romania; 5 Faculty of Medicine, Titu Maiorescu University, 040441 Bucharest, Romania; gabriel.gorecki@prof.utm.ro; 6 Filantropia Clinical Hospital Bucharest, 011132 Bucharest, Romania

**Keywords:** endometriosis, postmenopausal patients, dysmenorrheal, infertility, menopausal hormone therapy

## Abstract

Endometriosis is a chronic inflammatory disease, characterized by the presence of ectopic endometrial tissue, that leads to dysmenorrhea, painful intercourse and infertility. The shift in paradigm from the previous belief that endometriosis exclusively impacts women of reproductive age has brought attention to the condition in both premenarchal and postmenopausal women. Currently, 2–4% of postmenopausal women have endometriosis. Many women experience menopausal symptoms during the peri- and postmenopausal periods and require extensive investigations and monitoring in order to avoid the recurrence of endometriosis symptoms or the risk of malignant transformation when treatment with menopausal hormones is elected. Our goal was to compile and present a clear and concise overview of the existing literature on postmenopausal endometriosis, offering an up-to-date and precise summary of the available information.

## 1. Introduction

Endometriosis is largely known as a hormone-dependent condition of women in their reproductive years. This period usually begins at menarche or in the first years of adulthood. The cessation of follicular growth or ovulation at menopause is responsible for the hypo-estrogenic status that alleviates pain symptoms and leads to the regression of endometriosis. The persistence of disabling pain and other symptoms that occur in menopause may be the consequence of some ectopic endometrial tissue that remains active in the postmenopausal period. The occurrence of novo lesions after menopause has been described, but it is difficult to differentiate these lesions from pre-existent lesions that start becoming symptomatic only after menopause [1,2]. Many women in the postmenopausal period are found to have asymptomatic endometriosis lesions during the performance of pelvic imaging or surgical interventions for other conditions. The characteristics of endometriosis in menopausal women are summarized in Table 1.

## 2. Risk Factors

Endometriosis in postmenopausal patients is similar to endometriosis in premenopausal patients. It can manifest in diverse ways and might not be promptly identified or correctly diagnosed at first. A history of symptoms before menopause (including dysmenorrhea, chronic pelvic pain, dyspareunia, dyschezia and infertility) could be related to previously unrecognized endometriosis, and it should be considered as a part of diagnosing endometriosis in postmenopausal patients. Evidence of prior endometriosis in patients with a history of hysterectomy or pelvic surgery may include the presence of adhesions, difficulty in performing the procedure due to adhesions or distorted anatomy, with or without the presence of adhesions [3].

Hormone replacement therapy (HRT) has been associated with endometriosis in postmenopausal patients secondary to stimulation of the endometrial deposits by exogenous estrogen. This effect is even more expressed in unopposed estrogen therapy, in which the progestin component does not exist [4,5]. The risk of recurrence of endometriosis while using HRT is even higher in obese patients or in women with severe disease [6].

Immunohistochemical findings suggest that the presence of estrogen and progesterone receptors, along with CD10, in menopausal women supports the involvement of exogenous or endogenous estrogens in the development of postmenopausal endometriosis. This indicates the potential for reactivation of the disease when appropriate stimulation is provided to endometriotic lesions [7].

Obesity is another risk factor that may increase the serum estrogen levels, resulting in progression of the endometriosis lesions, findings on imaging and subsequent symptoms. Tamoxifen, a selective estrogen receptor modulator, is known to have an agonist effect on endometrial tissue and to be responsible for endometrial abnormalities, like hyperplasia, polyps and malignancy [8,9].

Additionally, there are documented cases of postmenopausal endometriosis where no clear source of systemic estrogen exposure or endogenous agonist activity can be identified [10]. Some researchers have proposed that, in certain patients, these cases may be attributed to the presence of aromatase within the endometriosis lesions, potentially leading to local estrogen production [5,11].

## 3. Pathophysiology

The presence of endometriosis in menopausal women may be explained by several pathogenetic mechanisms, but it is still considered unclear [12]. The pathophysiology of postmenopausal endometriosis is even more complex than that of premenopausal endometriosis.

**Figure 1:**
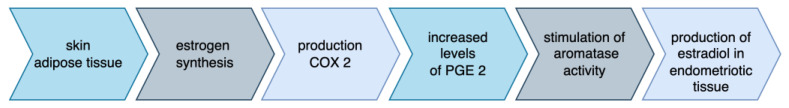
Local production of estradiol in endometriotic tissue.

Estrogen biosynthesis takes place mostly in the adipose tissue and the skin [13]. The expression of aromatase P450 has been demonstrated in several studies in both the eutopic and ectopic endometrium of premenopausal patients with endometriosis [14]. The production of the cyclooxygenase type 2 enzyme is induced by estrogen, leading to increased levels of prostaglandin E2, which is known as a stimulator of aromatase activity, suggesting a positive feedback loop that supports the ongoing local production of estradiol in endometriotic tissue [15]. This mechanism is shown in Figure 1. A relationship between endometriosis and epithelial ovarian cancer has been suggested in some studies. Zanetta et al. concluded, in a study on endometriotic women who developed cancer [16], that hyper-estrogenism (endogenous or exogenous) is the only risk factor for developing cancer in endometriotic lesions.

Some researchers have proposed the potential involvement of a deficient immune system in the development of endometriosis, which could facilitate the implantation and growth of ectopic endometrial cells. In postmenopausal women, there may be a relative state of immunosuppression, potentially enabling the establishment or progression of endometriotic lesions [17].

## 4. Symptomatology

Endometriosis in menopausal patients often manifests with non-specific clinical symptoms, like pelvic pain, ovarian cysts or intestinal symptoms. Due to their age, these patients are frequently suspected of having a potentially cancerous condition. An evaluation for malignancy is advisable to be made for all postmenopausal patients. Following menopause, the decline in estrogen levels in women with a history of endometriosis alleviates the symptoms associated with the condition. However, it may lead to the onset of distinct menopausal symptoms, like mood swings, hot flushes, night sweats and vaginal atrophy [18]. Complaints of pelvic pain frequently underestimate the severity of the disease in both pre- and postmenopausal cases of endometriosis.

In 2023, the United States Food and Drug Administration approved the use of a neurokinin 3 receptor antagonist (NK3R) called fezolinetant for menopausal vasomotor symptoms [19]. The decrease in estrogen levels in menopause causes increased neurokinin B signaling that activates NK3R followed by an increase in the level of the Gonadotropin-releasing hormone (GnRH) [20]. GnRH modulators represent one of the therapeutic options used in the treatment of endometriosis, and their effect is to suppress the hypothalamic–pituitary–gonadal axis. To modulate the axis, NK3R may represent a new therapeutic target [21]. This new drug may be useful in treating patients with vasomotor symptoms associated with endometriosis and requires studies to evaluate its effectiveness on endometriosis. Modulation of this neuroendocrine system can help to better understand endometriosis in menopause.

## 5. Localization

The most common localizations for endometriosis are the ovaries, utero-sacral ligaments and pelvic peritoneal surfaces [22]. Postmenopausal endometriosis is most frequently found at the level of ovaries [1,23], but it may also be localized in the urinary tract [24], stomach [25], large and small bowels [26,27], vagina [28], diaphragm [29], inferior vena cava [30] and skin [31]. The symptoms presented may be associated with the organ system where lesions are identified. Voiding dysfunction of the bladder or hematuria may be the result of lesions involving the bladder, whereas bowel obstructions or rectal bleeding may represent a consequence of lesions involving the bowel.

## 6. Clinical Examination and Diagnosis

Clinical examination, preoperative symptoms and the medical history of the patient represent a limited role for determination of the extension of endometriosis lesions. Furthermore, it is common to observe a discrepancy between the intensity of symptoms and the extent of lesions. Many patients may have severe lesions that remain asymptomatic. This factor significantly contributes to a delay of around 6 to 8 years from the initial onset of symptoms to diagnosis, both in premenopausal and postmenopausal patients. Pelvic vaginal and rectal examinations are useful for the identification of endometriosis nodules in the lower posterior compartment. However, it is important to note that clinical examinations may have normal results in a significant number of patients with deep infiltrating endometriosis [32]. Endometriosis continues to be a pathological condition that often experiences delayed diagnosis, particularly among older patients, due to the lack of available non-invasive methods for early-stage diagnosis. To date, the gold standard for diagnosing endometriosis and for obtaining histological confirmation of suspicious lesions is represented by laparoscopy and biopsy. Currently, no serum marker was found to be reliable for diagnosing endometriosis [33]. Diagnostic laparoscopy can be avoided because transvaginal ultrasound has progressed, and it is a very useful first-line imaging technique in the diagnosis of endometriosis. Ultrasound findings were described for women of menstrual age but also for menopausal women [34]. Magnetic resonance imaging (MRI) is the best non-invasive method for diagnosing endometriosis. The sensitivity and specificity of this method vary depending on the anatomic location and the number and dimensions of the lesions [35]. New diagnostic methods for debilitating diseases have been developed, and microRNA (miRNA) analysis has evolved. In 2022, a study was published that aimed to define the miRNA signature for endometriosis on saliva samples. This test is a useful non-invasive method for quick and early diagnosis [36]. Brulport et al. published a systematic review in 2024 about the biomarkers studied in endometriosis. The results showed that 1107 biomarkers were identified in nine biological compartments (peripheral blood, peritoneal fluid, cervical mucus, eutopic endometrium, ovaries, menstrual blood, urine, saliva and feces). Of these, only four biomarkers (TNF-a, TIMP-1, MMP-9 and miR-451) were identified in at least three of the mentioned tissues. Studies are needed to evaluate if these markers are also present in menopausal women with endometriosis and if they are useful for diagnosis [37].

## 7. Imaging and Findings

Laparoscopy is considered the gold standard for the diagnosis of endometriosis. However, non-invasive testing for the early diagnosis of endometriosis is preferred over laparoscopy, and it is important to note that no imaging technique can provide an absolute confirmation of endometriosis diagnosis, especially when it comes to peritoneal implants, where the results can be notably inconclusive. Several imaging techniques, such as transvaginal ultrasound scan, computerized tomography, rectal endoscopic sonography, magnetic resonance imaging (MRI) and 3D ultrasound, can be used to investigate deep infiltrating endometriosis [32].

Transvaginal sonography (TVS) has gained increased attention in recent years and is now being advocated as the primary investigative method for endometriosis because of its ability to allow for extensive exploration of the pelvis, cost efficient ratio, availability and the fact that it is well tolerated by patients [38]. TVS offers the advantage of avoiding radiation exposure and serves as the primary approach for assessing adnexal masses. However, its effectiveness in diagnosing other forms of endometriosis is somewhat restricted. Endometriomas have some distinct characteristics on ultrasound examination, typically appearing as unilocular cysts with a predominantly homogenous “ground glass” appearance. Discovering an endometrioma should be a signal to the clinician for the potential presence of moderate- to advanced-stage disease. It is important to note an important exception in postmenopausal women, where ovarian cysts displaying a “ground glass” appearance are linked to a 44% risk of malignancy [39]. Transvaginal sonography could play a role in evaluating conditions affecting the bladder and rectum.

In the presence of colon distension, computed tomography has a significant role in diagnosing bowel endometriosis. Patients with a history of endometriosis or chronic pelvic pain should be considered for genitourinary tract involvement when hydronephrosis or hydroureter are suggested by computed tomography [39].

Although it has limited indications in the diagnosis of endometriosis, MRI may be used as a non-invasive diagnostic method for deep infiltrating endometriosis. It has a high accuracy, offering the possibility to fully investigate the pelvic cavity [40]. Moreover, when the transvaginal ultrasound scan is not conclusive, MRI has been proven to be useful for diagnosing endometrioma in the presence of an adnexal mass. MRI can also be used to explore the involvement of the ureter and to evaluate the anatomy in the case of expanded pelvic adhesions [39].

## 8. Management

Surgical procedures with the removal of all visible endometriotic lesions should be performed in all postmenopausal patients with symptomatic endometriosis due to the high risk of recurrence and malignancy [41]. Medical therapy may be considered if there is a recurrence of pain after surgery or if surgery is not a viable option due to contraindications. Co-existing health conditions, such as advanced age or pelvic adhesions from prior surgeries, can also be reasons to exclude surgery [41,42]. It is estimated that around 12% of all endometriosis cases will eventually require a hysterectomy, with or without oophorectomy [43]. To prevent future recurrences, restore normal functioning of the bowel, urinary system or sexual organs, as well as alleviate pain, it is now recommended to remove all implants.

HRT remains controversial in postmenopausal patients who have undergone a surgical procedure on endometroid tissue. According to Zanetta et al., the prevalence of endometriosis in association with obesity or unopposed estrogen therapy represents an important risk factor for developing cancer in endometriotic lesions [16]. In premenopausal patients who undergo total hysterectomy with the removal of both ovaries and fallopian tubes due to endometriosis, HRT offers benefits that outweigh the risks. The initiation of hormone therapy in postmenopausal patients may cause an increasing risk of recurrence of endometriosis or malignant transformation [44].

The use of progesterone, whether administered orally or via an intrauterine system, has been suggested as a viable alternative treatment for patients who have contraindications for surgery. However, as of now, comprehensive data on this matter are lacking, and further studies are required to explore the effectiveness of progesterone in treating postmenopausal endometriosis [45].

Aromatase inhibitors function by reducing estrogen production outside the ovaries and inhibiting the feedback loop that amplifies inflammation and aromatase activity within endometriosis lesions. The primary concern associated with this treatment is the risk of osteoporosis and associated fractures. Aromatase inhibitors have a negative impact on bone mineral density, necessitating their use in conjunction with bisphosphonate therapy [46].

Postmenopausal patients with breast cancer may use tamoxifen as a hormonal substitution therapy because of it antiestrogenic effect. However, tamoxifen promotes endometriosis through unclear mechanisms [47]. Moreover, some cases of endometroid carcinoma or ovarian carcinoma were reported [48,49,50]. Due to limited data in the literature regarding a significant correlation between tamoxifen and malignant transformation, a possible coincidental association remains.

## 9. Malignant Transformation of Endometriosis Lesions

Patients with endometriosis have a higher risk of ovarian cancer (clear cell, endometrioid, low-grade serous type) [51,52]. The incidence of transformation from endometrioma to ovarian cancer is estimated at around 2–3% [53]. Another potential risk factor for malignancy is the increasing age of patients, particularly in the postmenopausal population. Early satiety, abdominal bloating and increasing abdominal growth may represent symptoms related to ovarian cancer in endometriosis. Bowel obstruction or pleural effusion is often seen due to advanced disease. Endometriosis-related malignancy has been described in the bowel, arising from deeply infiltrative lesions, leading to obstruction or bleeding [27].

The concept of atypical endometriosis was introduced, and it was defined as a precursor lesion for the appearance of clear-cell ovarian carcinoma and endometrioid ovarian carcinoma. A hyperestrogenic environment, oxidative stress, genetic mutations and cytokines can play a role in carcinogenesis and in the evolution from benign endometriosis to atypical endometriosis and then ovarian cancer [54]. The histological findings described cytological atypia (nuclear stratification, pleomorphism, hyperchromatism) and architectural atypia referring to hyperplasia. Careful histological evaluation and observation of changes beyond the diagnosis of endometriosis are important [55].

Distinguishing between benign and malignant tumors in postmenopausal women poses a challenge in terms of imaging features. Furthermore, some endometriosis lesions have a similar appearance to malignant lesions and may infiltrate adjacent tissues and organs. Somigliana et al. concluded that endometriosis is a pathological condition associated with the risk of any specific cancer [56].

Bertelsen et al. determined that the risk of developing breast cancer is approximately 0.97% when diagnosed with endometriosis at an age below 40 years. The elevated risk associated with endometriosis among postmenopausal patients might be due to some common risk factors between endometriosis in postmenopausal and breast malignancy or a dysfunctional endogenous estrogen [57].

An intermediate T2 signal with early avid enhancement and restricted diffusion, regardless of the localization, is often seen on MRI. Postcontrast T1-weighted image suppression and subtraction to examine enhancing mural nodules or septations in the case of a T1 hyperintense endometrioma represent specific considerations in the imagistic evaluation. Restricted diffusion may be helpful when it corresponds to concerning septations or nodules, but this might also be seen in benign endometriomas because of blood products [58]. As a result of increasing fluid produced by the tumor, the loss of T2 shading in malignant endometriomas might be helpful but non-specific [59]. Furthermore, an infiltrative appearance may be seen in extra-ovarian endometriosis-associated malignancy, but the features of malignancy may also be described in benign variants like polypoid endometriosis. Regarding bowel-invasive endometriosis, the T2 hyperintense “cap” of the mushroom is frequently lost in the case of a mucosal invasion. Also, the T2 hypointense fibromuscular stroma may be described as more intermediate in terms of signal. Glandular or cystic elements might remain in the setting of malignant transformation. Benign and malignant endometriosis often coexists regardless of localization [60].

## 10. Conclusions

Postmenopausal endometriosis is an underestimated type of endometriosis and may develop many years after menopause in the absence of an intake of estrogen or increased systemic production. The most common localization for postmenopausal endometriosis is represented by the ovaries. The pathogenic mechanism is still unclear. Endometriosis in postmenopausal women should be taken into consideration in the case of unclear pelvic pain, even without a history of endometriosis lesions. Treatment with exogenous estrogen has the potential to exacerbate the symptoms of endometriosis. Surgery is the main treatment modality. Aromatase inhibitors have a valid place in patients that have contraindications for surgery. Postmenopausal endometriosis has been associated with a higher risk of malignant transformation.

## Figures and Tables

**Table 1:** Characteristics of endometriosis in menopausal women.

Risk factors	History of symptoms before menopause
	Therapy with hormone replacement
	Obesity
	Therapy with tamoxifen
	Evidence of prior endometriosis
Symptomatology	Non-specific clinical symptoms
	Pelvic pain
	Intestinal symptoms
Localization	Ovaries
	Utero-sacral ligaments
	Pelvic peritoneal surfaces
	Other: urinary tract, stomach, large and small bowel, vagina, diaphragm, inferior vena cava and skin
Imaging	Transvaginal sonography
	Computerized tomography
	Magnetic resonance imaging (MRI)
Management	Surgical removal of all the visible endometriotic lesions
	Medical therapy (e.g., progesterone, aromatase inhibitors)

## Data Availability

Data sharing is not applicable to this article.
